# Investigating the Frequency and Outcome of Central Vein Sign and Paramagnetic Rim Lesions in Children With MOGAD

**DOI:** 10.1212/WNL.0000000000214410

**Published:** 2026-01-09

**Authors:** Riccardo Nistri, Laura Cacciaguerra, Simone Sacco, Akash Virupakshaiah, Ermelinda De Meo, Nico Papinutto, Roland G. Henry, Hadas Meirson, Cheryl Hemingway, Thomas Rossor, Evangeline Wassmer, Liat Bensira, Li-tal Pratt, Asthik Biswas, Sniya Sudhakar, Kshitij Mankad, John J. Chen, Sean J. Pittock, Frederik Barkhof, Olga Ciccarelli, Emmanuelle Waubant, Eoin P. Flanagan, Yael Hacohen

**Affiliations:** 1 Queen Square MS Centre, Department of Neuroinflammation, UCL Queen Square Institute of Neurology, Faculty of Brain Sciences, University College London, United Kingdom;; 2 Pediatric Neurology, Great Ormond Street Hospital for Children, London, United Kingdom;; 3 Department of Neurology and Mayo Clinic Center for Multiple Sclerosis and Autoimmune Neurology, Mayo Clinic, Rochester, MN;; 4 Weill Institute for Neurosciences, Department of Neurology, University of California, San Francisco;; 5 Pediatric Neurology Institute, Dana-Dwek Children's Hospital, Tel Aviv Sourasky Medical Center, Israel;; 6 Sackler Faculty of Medicine, Tel Aviv University, Israel;; 7 Children's Neurosciences, Evelina London Children's Hospital, Guy's and St Thomas' NHS Foundation Trust, King's Health Partners Academic Health Science Centre, United Kingdom;; 8 Department of Neurology, Birmingham Children's Hospital, United Kingdom;; 9 Pediatric Radiology, Imaging Division, Tel Aviv Sourasky Medical Center, Israel;; 10 Department of Neuroradiology, Great Ormond Street Hospital, Great Ormond Street Hospital Trust, London, United Kingdom;; 11 Department of Ophthalmology, Mayo Clinic, Rochester, MN;; 12 Department of Radiology & Nuclear Medicine, Amsterdam UMC, Vrije Universiteit, the Netherlands;; 13 NIHR University College London Hospitals Biomedical Research Centre, United Kingdom; and; 14 Department of Neuroinflammation, National Hospital for Neurology and Neurosurgery, University College London Hospitals NHS Foundation Trust, United Kingdom.

## Abstract

**Background and Objectives:**

Central vein sign (CVS) is a common feature in multiple sclerosis (MS) lesions, but its frequency in myelin oligodendrocyte glycoprotein antibody-associated disease (MOGAD) varies significantly across studies. Paramagnetic rim lesions (PRLs) are described in MS, but not in MOGAD. Our goals were to evaluate the prevalence of PRLs and CVS in a large multicenter cohort of pediatric MOGAD. We compared CVS frequencies between acute vs remission phases, as well as with longitudinal dynamics of lesion evolution.

**Methods:**

In this longitudinal retrospective multicenter study, clinical MRIs were assessed from pediatric patients with MOGAD, who had (1) ≥1 brain lesion, (2) susceptibility-based imaging (SBI), and (3) follow-up MRI at least 3 months apart. T2-weighted and fluid-attenuated inversion recovery sequences were analyzed for lesion detection and resolution. SBI was used to assess CVS and PRLs following North American Imaging in MS Cooperative criteria.

**Results:**

A total of 65 patients and 130 scans were included. A total of 520 lesions were evaluated. The most common reason for lesion exclusion was size (n = 143, large confluent lesions, n = 122, <3 mm, n = 21). CVS was detected in 97 of 327 (29.7%) lesions, with 32 of 65 (49.2%) patients having at least 1 CVS+ lesion. Patients with ≥1 CVS+ lesion (32/65, 49.2%) had a higher number of lesions (*p* = 0.002) and lesions suitable for CVS analysis (*p* < 0.001). Of the 31 patients with ≥3 brain lesions, 11 of 31 (35.5%) had >40% CVS+ lesions and 7 (22.5%) had >50% CVS+ lesions. Only 4 patients had ≥6 CVS+ lesions. The proportion of CVS+ lesions was lower in patients who had SBI acquired during an acute attack vs patients scanned during remission (16% vs 33%, *p* = 0.015). The rate of lesion resolution was higher in CVS− lesions (177/230, 76%) compared with CVS+ (42/97, 42%, *p* < 0.001). No PRLs were identified.

**Discussion:**

Lesion pathobiology in MOGAD is heterogeneous. CVS identified persistent rather than transient lesions, with resolution more common among CVS− lesions. The high frequency of confluent or multivein lesions limited the proportion suitable for CVS analysis. MRI timing influenced CVS detection, which was higher in remission, suggesting that time of acquisition contributes to variability across MOGAD studies. No PRLs were found, supporting their potential as biomarkers distinguishing MOGAD from MS.

## Introduction

Myelin oligodendrocyte glycoprotein antibody-associated disease (MOGAD) is a recently identified demyelinating disorder distinct from multiple sclerosis (MS).^1,2^ A growing body of research has been focusing on the usefulness of MRI to distinguish MOGAD from pediatric-onset MS (POMS).^1^ Longitudinal conventional and advanced cross-sectional MRI imaging seem to have the best diagnostic performances to distinguish the 2 conditions.^3,4^ Assessing imaging differences between MOGAD and MS is, however, extremely important, not only for differential diagnosis, but also to unveil in vivo some of the potential pathologic differences between the 2 diseases.

Conventional brain imaging in MOGAD can vary significantly, ranging from normal findings to subtle changes in white matter (WM) tracts or large, ill-defined WM lesions, with or without grey matter involvement, with some degree of overlap with MS.^5,6^ Longitudinal studies in pediatric populations showed, however, that brain WM lesions in MOGAD evolve differently over time when compared with MS. In fact, in MOGAD, acute T2 hyperintense lesions often resolve on follow-up MRI scans, while this is extremely rare in MS.^4,7^ The resolution of most lesions in MOGAD might suggest that some of the MRI changes detected in the acute phase may be related to transitory phenomena rather than demyelination.^8,9^

Several cross-sectional studies have already assessed the role of advanced MRI sequences in discerning MOGAD from POMS. Susceptibility-based imaging (SBI, in the form of susceptibility-weighted imaging, susceptibility weighted angiography, or 3-dimensional echoplanar imaging) has emerged as a potential tool to cross-sectionally differentiate MS from MOGAD due to the higher frequency of central vein signs (CVSs) observed in MS lesions.^10,11^

Aside from the assessment of perivenular demyelination, SBI has unveiled other potential pathologic differences between MS and MOGAD. SBI in MS allows assessment of paramagnetic rim lesions (PRLs), a specific type of WM lesion that has been associated pathologically with chronic demyelination. Such lesions are currently considered highly specific for MS^12-14^ and have never been observed in MOGAD. The lack of PRLs in MOGAD might suggest that chronic inflammation and ongoing demyelination at edge of lesions do not occur in this disease.

With the current multicenter retrospective study, we aim to evaluate the prevalence of PRLs and CVS in our large multicenter cohort of pediatric MOGAD following published guidelines for identifying CVS and PRL in patients with MS.^15,16^ Given the dynamic and transient nature of MRI changes in MOGAD, we hypothesize that the frequency of CVS might differ when a scan is obtained in the acute vs remission phases. Moreover, the persistence of CVS lesions in MOGAD may indicate perivenular demyelination rather than transient changes, and therefore, this may become a prognostic factor that predicts lesion persistence in this population, and future studies are needed to confirm it.

## Methods

This was a multicentric retrospective study including patients from 6 tertiary neuroimmunology referral centers from 3 countries: the United Kingdom (n = 3), the United States (n = 2), and Israel (n = 1). Pediatric onset patients (i.e., younger than 18 years of age) who fulfilled the international diagnostic criteria for MOGAD^17^ and had neuroimaging performed between January 2014 and August 2024 were included. All patients had (1) at least 1 brain lesion, (2) at least 1 clinical MRI scan including SBI, and (3) a follow-up MRI scan performed at least 3 months after reference/initial scan for the evaluation of lesions dynamic (eFigures 1 and 2). T2-weighted and fluid-attenuated inversion recovery (FLAIR) sequences were used for lesion identification and to assess overtime lesion evolution, while SBI was used for CVS identification and the assessment of PRLs. Postcontrast T1-weighted images were analyzed as well for the presence of enhancing lesions and to exclude the acute nature of lesions showing paramagnetic rim. Patients with coexisting neuroinflammatory disorders (e.g., anti-NMDA receptor encephalitis) were excluded. Acute attacks were defined as “new neurologic symptom” or “clear acute worsening of previous neurologic deficits” with objective clinical signs, lasting for at least 24 hours and attributed to an inflammatory CNS event with relapses defined as occurring after a period of clinical remission of >1 month, as defined by the International MOGAD Panel proposed criteria,^17^ confirmed by the treating physician. MRI scans were stratified in 2 groups: (1) acute scans performed, while the child is symptomatic within 6 weeks from symptoms onset, (2) remission scans performed at the time of clinical remission and at least 6 weeks from symptom onset.^17^

### Data Collection

Clinical data including demographics, clinical features (i.e., types and timing of attacks, treatments, and expanded disability status scale [EDSS]) as well as laboratory results were retrospectively reviewed from patients' electronic medical records.

### MRI Analysis

Thirty-seven MRI scans were obtained on 1.5T and 28 on 3T. The MRI acquisition protocol is detailed in eTable 1.

T2-hyperintense lesions were identified on T2-FLAIR images, and the presence of CVS was assessed on coregistered SBI. The evaluation of CVS and paramagnetic rim was performed by consensus of 2 raters per center (either 2 neurologists with at least 5 years of expertise in neuroimaging or 2 neuroradiologists or 1 neurologist and 1 radiologist) blinded to the participant's clinical data, following the North American Imaging in MS Cooperative guidelines.^15^ Large confluent lesions containing more than 1 vein, punctate WM lesions (<3 mm), and lesions with technical signal artifacts were excluded.^15^ Lesions were classified as periventricular (with 1 edge in contact with a ventricle), deep WM, and juxtacortical (with 1 edge in contact with the cortex) or other locations. The presence of enhancement was evaluated on postcontrast T1-weighted sequences.

### Statistical Analysis

Statistical analyses were conducted using SPSS, version 29 for Windows (IBM Corp., Armonk, NY) and RStudio version 2024.09.0+375. Continuous variables were compared using the Mann-Whitney *U* test according to the normality assumption, while categorical variables were compared with the χ^2^ test or Fisher exact test based on the number of observations. A *p* value of <0.05 was considered significant. The associations between the resolution of all lesions and patients' clinical-radiologic features were investigated through univariable binary logistic regressions.

### Standard Protocol Approvals, Registrations, and Patient Consents

This study was approved by the Great Ormond Street Hospital Research and Development Department (reference: 16NC10), Mayo Clinic Institutional Review Board (IRB) (IRB 08-006647), the UCSF IRB (IRB 23-38563), and Tel Aviv Sourasky medical center ethics board (IRB 0523-23).

### Data Availability

For purposes of replicating procedures and results, any qualified investigator can request anonymized data from the corresponding author, subject to ethics clearance and approval by all authors and their respective sites.

## Results

### Study Population

Sixty-five children fulfilled the inclusion criteria. Patients' demographics are summarized in Table 1. Thirty-seven patients were female (56.9%), and the median age at the time of first SBI scan was 6 years (interquartile range [IQR] 4.4–11.6). Time from symptom onset to SBI MRI scan was 25 days (IQR 4–149).

**Table 1:** Comparison of Clinical and Imaging Features of Patients With Reference/Initial MRI Acquired During the Acute Phase vs Remission

	Acute	Remission	*p* Value
No. of patients	42	23	0.920
Age at the time of MRI, y, median (IQR)	5.6 (4–8.75)	11 (6–14)	0.0064
Sex (F:M)	23:19	14:9	0.831
Duration from symptoms onset to acute scan, d, median (IQR)^a^	7 (1.25–24.75)	NA	
Disease duration from last relapse, d, median (IQR)	NA	353 (129.75–483.5)	
No. of previous relapses, median (IQR)	0 (0–1)	1 (0–3)	0.006
Duration from symptoms onset to steroid treatment, d, median (IQR)	3.5 (0–7.25)	NA	
Duration from steroid treatment to reference/initial MRI, d, median (IQR)	0 (0–2.75)	NA	
Total no. of WM lesion, median (IQR)	6 (2.25–9.5)	4 (2–5)	0.09
% Lesions excluded from CVS analysis	151/411 (36.7)	42/109 (38.5)	0.816
% Lesions suitable for CVS analysis	260/411 (63.3)	67/109 (61.5)	0.815
% CVS+ lesions, median (IQR)	16.7 (0–50)	33.3 (0–66.7)	0.015
% CVS+ in patients with at least 3 lesions suitable for CVS analysis, median (IQR)	27 (15.5–46.4)	33 (8.3–54.3)	0.293

Abbreviations: CVS = central vein sign; CVS+ = central vein sign positive; F = female; IQR = interquartile range; M = male; WM = white matter.

a Duration from symptoms onset to acute scan applies for disease onset (39 cases) and for the 4 patients who were acquired during a relapse, where it refers to their most recent clinical events.

Patients presented with acute disseminated encephalomyelitis (n = 39), optic neuritis (n = 12), encephalitis (n = 10), and transverse myelitis (n = 4). In 42 patients, the reference/initial scan including SBI was performed during an acute attack (either first attack, n = 38, or a subsequent attack (or relapse) n = 4), at a median of 7 days from symptom onset (IQR 1.3–24.8). In 23 patients, the reference/initial scan was performed during remission at a median of 11.5 months from the last clinical attack (IQR 5–23.3). At follow-up (median 18 months, IQR 6–30), 32 patients (49.2%) had a relapsing disease course, and the median EDSS was 0 (IQR 0–1).

### Identification of Lesions Suitable for CVS Assessment

**Figure 1:**
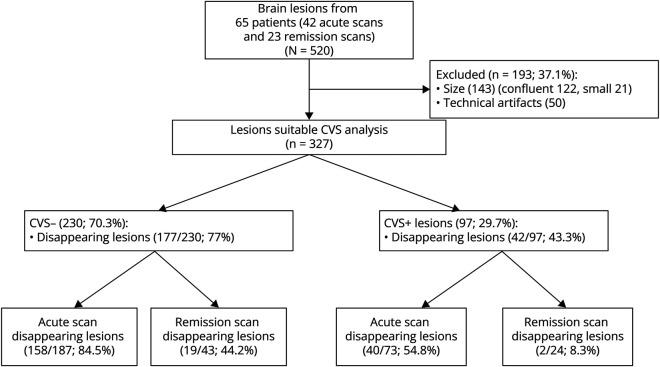
Flowchart of Lesion Selection and Stability Analysis in Pediatric MOGAD Flowchart summarizing lesion analysis in CVS assessment. Of 520 lesions, 193 (37.1%) were excluded, leaving 327 lesions suitable for CVS analysis. Among these, 230 (70.3%) were classified as CVS-negative, with 177 of 230 (77%) disappearing lesions. Of the CVS-negative lesions, 158 of 187 (84.5%) disappeared on acute scans, emphasizing a high rate of resolution during the acute phase, while 19 of 43 (44.2%) disappeared on follow-up scans. By contrast, 97 (29.7%) lesions were classified as CVS-positive, with 42 of 97 (43.3%) disappearing lesions. Among CVS-positive lesions, 40 of 73 (54.8%) disappeared on acute scans, suggesting a moderate resolution rate during the acute phase, and 2 of 24 (8.3%) disappeared on follow-up scans. CVS = central vein sign; MOGAD = myelin oligodendrocyte glycoprotein antibody-associated disease.

**Figure 2:**
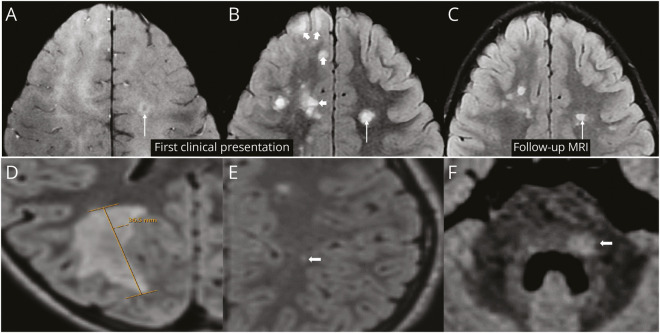
Persisting and Resolving Lesions Axial SWI (A) and FLAIR (B) images reveal multiple focal lesions in the white matter. Among these, a lesion in the left frontal deep white matter (long arrow, B) demonstrates the CVS on SWI (long arrow, A), while the remaining lesions (small arrows, B) lack the CVS. Follow-up imaging shows the resolution of most CVS-negative lesions, with the CVS-positive lesion (arrow, C) persisting. D, E, and F show examples of lesions excluded from analysis. Axial FLAIR images show a large confluent lesion >2 cm in size (D), a punctate lesion <3 mm in size (arrow, E), and a brainstem lesion (arrow, F). CVS = central vein sign; FLAIR = fluid-attenuated inversion recovery; SWI = susceptibility-weighted imaging.

A total of 130 scans and 520 lesions were evaluated of which 327 (62.9%) were suitable for the analysis, while 193 were excluded as per the above-mentioned criteria. The lesions that needed to be excluded more frequently were bigger, confluent and often with multiple veins within it (n = 143, 7 of which were also infratentorial), followed by lesions smaller than 3 mm (n = 21). Fifty additional lesions were excluded because of technical artifacts (32 of which were infratentorial). Figure 1 summarizes the inclusion and exclusion processes with imaging examples illustrated in Figure 2. The median number of T2/FLAIR lesions per patient was 4 (IQR 2–8), with a median of 2 (IQR 1–4) lesions excluded and 2 (IQR 1–6) lesions suitable for CVS evaluation.

### CVS and PRLs at the Reference/Initial MRI Scan

Among the 327 lesions suitable for CVS analysis, 97 (29.7%) were CVS+. Of CSV+ lesions, 41 (42.2%) were juxtacortical, 28 (28.9%) were in the deep WM, and 28 (28.9%) were periventricular.

The median CVS+ count per patient was 1 (IQR 0–2), with 32 of 65 (49.2%) patients having at least 1 CVS+ lesion. No PRL was detected in any of the patients.

**Figure 3:**
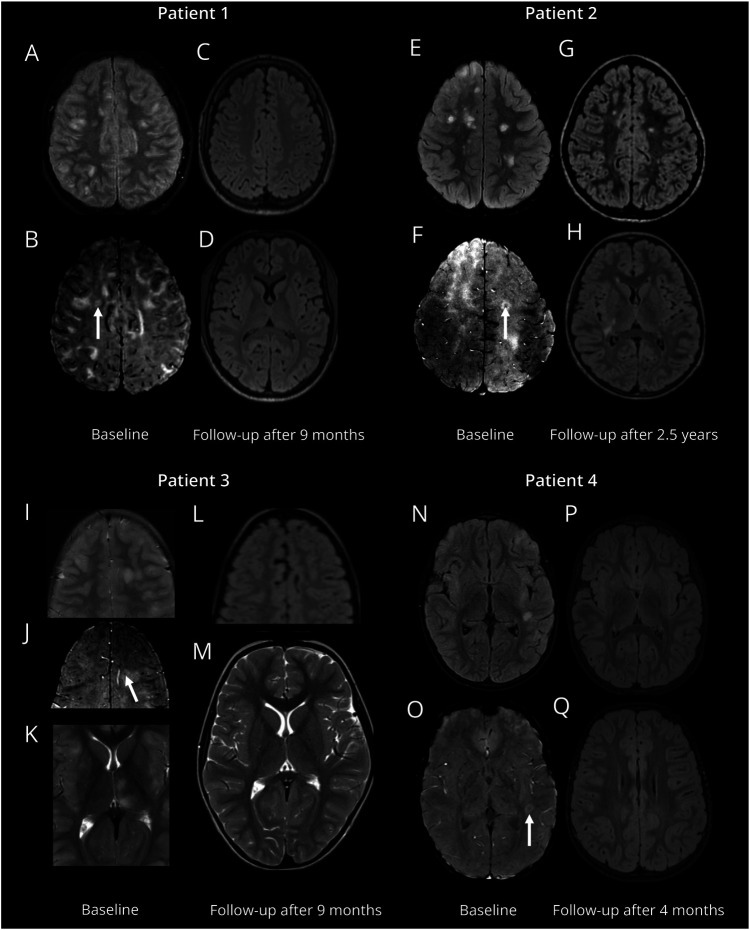
Imaging Findings in Patients With ≥6 CVS+ Lesions MRI scans from 4 patients (P1, P2, P3, P4) demonstrating reference/initial scan and follow-up evaluations. P1: Reference/initial scans include T2-weighted imaging (A) and SWI (B), with a white arrow highlighting a CVS+ lesion. Follow-up scans 9 months after (C, D) show complete resolution of the lesion. P2: Reference/initial scans include T2-weighted imaging (E) and SWI (F), with a white arrow pointing to a CVS+ lesion. Note the presence of thalamic lesions (K). Follow-up scans 2.5 years after (G, H) show persistence of CVS+ lesion. P3: Reference/initial scans include T2-weighted imaging (I, K) and SWI (J), follow-up scans 9 months after (L, M) demonstrate complete resolution of all lesions. P4: Reference/initial scans include T2-weighted imaging (N) and SWI (O), with a white arrow marking a CVS+ lesion. Follow-up scans 4 months after (P, Q) show resolution of the lesion. CVS = central vein sign; SWI = susceptibility-weighted imaging.

Of the 31 patients with ≥3 brain lesions, 11 of 31 (35.5%) had >40% CVS+ lesions and 7 had >50% CVS+ lesions. Four patients had ≥6 CVS+ lesions, with 3 also having >40% CVS+. All authors reviewed the diagnosis of the 12 patients who fulfilled the 40% rule or had ≥6 CVS+ lesions, and all independently agreed these patients had MOGAD. eTable 2 summarizes key clinical features with imaging findings in Figure 3. When evaluating these rules using the total number of lesions, 47 patients had ≥3 brain lesions, 6 fulfilled the 40% rule, and 3 fulfilled the 50% rule.

### CVS Lesions in Acute vs Remission MRI Scans

The proportion of CVS+ lesions was lower in patients who had their SBI MRI during an acute attack vs patients scanned during remission (16% vs 33%, *p* = 0.015). Patients who were scanned during remission were older, but there were no other differences in patients' demographics, disease phenotype, number of previous relapses, or total T2 lesion load between the 2 groups (Table 1).

### CVS in Persisting and Disappearing Lesions

Lesion dynamics were evaluated by comparing all the reference/initial T2-FLAIR images with those of a follow-up MRI scan acquired after a median of 18 months (IQR 6–30). Of the 327 lesions evaluated for CVS analysis, 219 (67%) resolved at follow-up imaging (Figure 2). The rate of lesion resolution was higher in CVS− lesions (177/230, 77%) compared with CVS+ lesions (42/97, 43.3%, *p* < 0.001). Relapsing phenotype at the last follow-up was associated with a reduced likelihood of lesion resolution. All other parameters were not associated with lesion resolution (eTable 3). In 38 patients with a follow-up scan acquired after 12 months from the SBI scan, 137 of 204 (67.2%) lesions resolved vs 82 of 123 (66.7%) with follow-up of less than 12 months (*p* = 0.999). No difference was seen in the CVS+ lesion resolution rate between the 2 groups (34/54 vs 21/43, *p* = 0.235).

### CVS in Persisting and Disappearing Lesions in Acute Scans

Forty-two acute scans with a total of 260 lesions were suitable for CVS analysis. Of these 198 (76.2%) resolved on follow-up imaging. The rate of lesion resolution over a median follow-up period of 20 months (IQR 9–36) was higher in CVS− lesions (158/187, 84.5%) compared with CVS+ lesions (40/73, 54.7%, *p* < 0.001).

Thirty-eight acute patients had a follow-up scan conducted at least 6 months after the acute scan (median follow-up 22.7 months [IQR 12–37.7]). In these patients, a total of 235 lesions were suitable for CVS analysis and 178 (75.7%) resolved at follow-up. The rate of lesion resolution was higher in CVS− lesions (145/173, 83.8%), compared with CVS+ lesions (33/62, 53.2%, *p* < 0.001).

### Clinical and Imaging Features of Acute Patients With Monophasic vs Relapsing Phenotype

Of the 38 patients with a scan acquired acutely at onset, 16 (42%) had at least another relapse. No differences were found between the 2 groups at onset (Table 2). Monophasic patients had a higher number of lesions resolving at last follow-up (88/110, 80% vs 66/101, 65.3%, *p* = 0.025).

**Table 2:** Comparison of Clinical and Baseline Imaging Features of Acute Patients With a Relapsing or a Monophasic Phenotype

	Relapsing (n = 16)	Monophasic (n = 22)	*p* Value
Age at MRI, y, median (IQR)	5 (4–8.25)	5.5 (4–8.7)	0.876
Sex (F:M)	5:11	15:7	0.055
Disease duration at first MRI, d, median (IQR)	8.5 (0.75–18.75)	5 (0.5–16)	0.975
Time onset to steroid treatment, d, median (IQR)	5 (1–9)	3 (0–7)	0.910
Presenting phenotype			
ADEM	12	16	0.998
Cortical encephalitis	2	4	0.981
ON	2	1	0.562
TM	0	1	0.999
CSF analysis			
Raised cell count (WCC >5)	10/16	15/22	0.985
Raised protein (>0.4 g/L)	4/16	5/22	0.999
Positive oligoclonal bands	3/16	3/22	0.994
CSF MOG antibodies/total tested	2/4	4/5	0.813
MRI analysis			
Evidence of enhancement (>GAD+ lesion)	5/13	4/21	0.397
No. of lesions per scan, median (IQR)	7.5 (2–11.5)	6 (3–8)	0.799
Proportion of lesions suitable for analysis, n (%)	101/157 (64.3)	110/194 (56.7)	0.180
No. of lesions suitable for CVS analysis, median (IQR)	4 (0.75–7)	2 (1–6)	0.968
No. of CVS+ lesions, median (IQR)	1 (0–2)	1 (0–1)	0.908
Follow-up MRI and clinical outcome			
Total follow-up time, mo, median (IQR)	29 (15.2–39.3)	14 (6–28.5)	0.060
Time reference/initial scan to follow-up scan, mo, median (IQR)	29 (13.5–39)	14 (6–28.5)	0.066
Normal MRI at last follow-up	2/16	8/22	0.202
% lesions resolved at last follow-up	66/101 (65.3)	88/110 (80)	0.025
EDSS at last follow-up, median (IQR)	0 (0–1)	0 (0–1)	0.999

Abbreviations: ADEM = acute disseminated encephalomyelitis; CVS = central vein sign; CVS− = central vein sign negative; CVS+ = central vein sign positive; EDSS = expanded disability status scale; F = female; IQR = interquartile range; M = male; MOG = myelin oligodendrocyte glycoprotein; ON = optic neuritis; TM = transverse myelitis; WCC = white blood cell count.

### Clinical and Imaging Features of Patients With and Without at Least 1 CVS+ Lesion on Reference/Initial MRI Scan

Patients with at least 1 CVS+ lesion had a higher number of lesions (*p* = 0.002) and a higher number of lesions suitable for CVS analysis (*p* < 0.001). Median time between MRI1 and MRI2 was 21.2 (IQR 8.5–36.25) months. Despite similar clinical outcomes, CVS− patients had a higher number of lesions resolving (*p* < 0.001) at the follow-up MRI scan. Disease duration at the time of the SBI scan was not different between patients with or without CVS+ lesions, with the first group having a median of 1 month (IQR 0–18.5) vs a median of 0 months (0–4; *p* = 0.233). No additional differences emerged. Further details are outlined in Table 3.

**Table 3:** Comparison of Clinical and Baseline Imaging Features of Patients With and Without at Least 1 CVS+ Lesion on Reference/Initial MRI Scan

	CVS+ (n = 32)	CVS− (n = 33)	*p* Value
Age at MRI, y, median (IQR)	6 (4.7–12)	6.5 (4/11)	0.893
Sex (F:M)	18/14	19/14	0.998
Acute MRI, n (%)	20 (62.5)	22 (66.7)	0.980
Disease duration at first MRI, mo, median (IQR)	1 (0–18.5)	0 (0–4)	0.233
Previous relapse, median (IQR)	0 (0–3)	0 (0–1)	0.194
Presenting phenotype			
ADEM	19	20	0.999
Cortical encephalitis	5	5	0.999
ON	6	6	0.999
TM	2	2	0.999
CSF analysis			
Raised cell count (WCC >5)	14/32	17/33	0.705
Raised protein (>0.4 g/L)	7/32	6/33	0.999
Positive oligoclonal bands	3/32	4/33	0.999
CSF MOG antibodies/total tested	4/5	4/8	0.565
MRI analysis			
Evidence of enhancement (>GAD+ lesion)	5/25	7/30	0.900
No. of lesion per scan, median (IQR)	7 (4–13.5)	3 (1–5)	0.002
Proportion of lesions suitable for analysis, n (%)	242/347 (69.7)	85/173 (49.1)	0.001
No. of lesions suitable for CVS analysis, median (IQR)	5.5 (3–9)	1 (0–2)	<0.001
Follow-up MRI and clinical outcome			
Total follow-up time, mo, median (IQR)	34.5 (15.2–66.4)	20.2 (7.8–38)	0.122
Time reference/initial scan to follow-up scan, mo, median (IQR)	21.2 (8.5–36.25)	12.2 (3–25)	0.437
Normal MRI at last follow-up	6/32	10/33	0.555
% lesions resolved at last follow-up	156/242 (64.5)	63/85 (74.1)	0.135
Relapsing phenotype at last follow-up	18/32	14/33	0.453
EDSS at last follow-up, median (IQR)	0.5 (0–1.5)	0 (0–1)	0.390

Abbreviations: ADEM = acute disseminated encephalomyelitis; CVS = central vein sign; CVS− = central vein sign negative; CVS+ = central vein sign positive; EDSS = expanded disability status scale; F = female; IQR = interquartile range; M = male; MOG = myelin oligodendrocyte glycoprotein; ON = optic neuritis; TM = transverse myelitis; WCC = white blood cell count.

## Discussion

CVS and PRLs are increasingly recognized as valuable imaging biomarkers for distinguishing MS from other demyelinating diseases, as reflected by their proposed inclusion in the forthcoming revision of the McDonald criteria. This growing emphasis makes it essential to better characterize the frequency and relevance of these imaging features in large cohorts of patients with MOGAD, particularly in relation to clinical presentation and lesion evolution. In this multicenter, retrospective study of a large pediatric MOGAD cohort, we aimed to address 3 key objectives: (1) the applicability of international consensus guidelines published in MS for assessing CVS and PRLs in MOGAD, (2) potential differences in the prevalence of CVS+ lesions between acute and remission MRI scans, and (3) the relationship between CVS status and lesion persistence over time. Our analyses yield several novel insights that enhance understanding of MOGAD pathophysiology and its distinction from MS. Most notably, we found a complete absence of paramagnetic rims among more than 500 detected brain WM lesions, suggesting a much lower propensity for chronic active lesions in MOGAD compared with MS.^18^ Furthermore, we demonstrate that CVS− lesions are more likely to resolve than CVS+ lesions, supporting the hypothesis that CVS+ lesions reflect perivenular demyelination, whereas CVS− lesions may arise from transient pathologic processes such as edema or reversible inflammatory changes.^3,19^

Approximately half of our cohort had at least 1 CVS+ lesion. These patients had a higher overall lesion burden and more lesions suitable for CVS assessment, but did not differ from CVS− patients in other clinical or radiologic characteristics, suggesting that the presence of CVS+ lesions does not define a distinct pathobiological subgroup within MOGAD. Notably, a lower proportion of CVS+ lesions was observed on acute-phase MRI compared with remission scans. This trend likely reflects lesion dynamics; CVS+ lesions tended to persist over time, whereas CVS− lesions were more likely to resolve, thereby influencing the proportion of CVS+ lesions detected at different time points. When focusing on the acute phase subcohort of 42 patients, CVS− lesions showed a higher tendency toward resolution (84.5% vs 54.7% for CVS+ lesions). This trend further supports our main finding that CVS status may serve as an indicator of lesion persistence. This observation may help explain the wide range of CVS+ lesion rates reported across MOGAD studies.^20,21^ An increase in the proportion of CVS+ lesions over time has also been reported in MS, where rates rose from 67% to 82%^22^; however, in MS, this phenomenon is believed to result from transient edema within acute contrast-enhancing plaques that initially obscures the central vein, rather than from lesion resolution.

Although both MS and MOGAD lesions show the coexistence of perivenular and nonperivenular plaques, their frequency, evolution, and pathologic characteristics differ.^23^ The radiologic disappearance of lesions in MOGAD, particularly frequent among CVS− lesions, suggests a pathologic substrate different from demyelination and gliosis, likely related to transitory edematous changes. MOGAD pathologic studies are more likely to be biased toward atypical presentation, and lesional biopsies may not represent the full spectrum of MOGAD lesions, with their biology being heterogeneous even within the same patient and likely including both focal demyelination and transitory edematous changes.

Although CVS has been proposed as a diagnostic marker for differentiating MS from other CNS inflammatory diseases, in our cohort, 4 patients met the “rule of 6,”^24^ 11 met the “40% rule,”^15^ and 7 (10.6%) met the “50% rule.”^25^ Given these unexpectedly high rates of CVS+ lesions, we conducted a rigorous review of these cases. Patients with more than 40% CVS+ or more than a total of 6 CVS+ lesions were independently reviewed by all authors to confirm their diagnosis (eTable 2). As per the MOGAD diagnostic criteria,^17^ patients with no titers available had additional supporting features. This thorough validation process strengthens our findings, which align with previous studies where 7.1% of MOGAD cases met the 50% threshold.^20^ Only 4 patients in our study had at least 6 CVS+ lesions, 1 with only 27% CVS+ lesions, mirroring MS-like values. Studies show that a greater proportion of patients with MS meet the 40% and 50% CVS criteria compared with patients with MOGAD,^13,26^ affirming CVS as a valuable diagnostic tool for differentiating between MS and MOGAD.^14^ However, our data demonstrate that meeting these criteria does not definitively rule out MOGAD diagnosis. The variable evolution of lesions in patients with MOGAD can easily modify the CVS values, making the differential diagnosis challenging only on the base of this method.

It is important to note, however, that our study confirms the usefulness of SBI and warrants its acquisition for the assessment of pediatric demyelinating disorders. The absence of PRLs in our large multicenter MOGAD cohort, once again confirms the recent evidence that these lesions might represent a feature highly specific to MS.^12^ Although earlier studies suggested 3T was superior to 1.5T in detecting PRLs, recent studies suggested the detection rate is similar in both fields.^27,28^ Combining the assessment of CVS (highly sensitive for MS but not completely specific) and PRLs (highly specific but not sensitive) is key to maximizing the usefulness of SBI in differentiating MOGAD over MS. Our findings on PRLs and CVS contrast with the recently proposed MS diagnostic criteria for pediatric patients, which exclude PRLs but include a CVS+ lesion cutoff for MS diagnosis. Although no direct comparison was made with an MS cohort, in keeping with previous publications of PRLs seen in most of the pediatric patients with MS,^13,14^ no PRLs were identified in this MOGAD cohort.

Our study has several limitations. First, its retrospective design and use of MRI scans acquired on different scanners across multiple centers introduce variability. Although CVS assessment methods have been validated in adults, their application in pediatric populations, particularly in MS and MOGAD, remains less well-established. This raises challenges in interpreting CVS in pediatric MOGAD, including notable interrater variability.^29^ To mitigate this, we used a standardized processing methodology, with coregistration of FLAIR and susceptibility-based images, to optimize CVS detection. Second, the relatively short MRI follow-up period may have limited our ability to capture lesion resolution over longer timescales, suggesting that CVS could be more accurately considered a marker of prolonged, but not necessarily permanent, lesion persistence.

The large confluent lesions typically seen in pediatric MOGAD^30,31^ resulted in specific technical challenges and artifacts, and indeed, 193 lesions (36.8%) were excluded. An additional limitation was the lack of CVS evaluation at 2 distinct timepoints, which prevented us from assessing the effect of edema during the acute phase on CVS detection. This phenomenon can have influenced our results on lesion persistence and needs further investigations. Besides, in our study, no interrater evaluation was performed because scans were evaluated together.

Finally, our cohort had a higher relapse rate (approximately 50%) compared with the 20% relapse^32^ rate typically reported in pediatric MOGAD cohorts. This likely reflects a selection bias toward patients seen at tertiary referral centers, who are more likely to have relapsing disease and undergo multiple MRI scans with advanced sequences. Although these patients represent just a minority of the patients seen in our centers (eFigure 1), this subgroup represents an ideal population for investigating lesion dynamics over time.

Our findings offer potential new insights into the biology of CVS+ lesions in MOGAD. The CVS is likely related to a perivenular distribution of focal WM demyelination, which, being particularly frequent in MS, results in an average CVS+ rate of around 80% in most of the studies.^22,33^ Although it is known from pathologic studies that even MOGAD can present perivenular demyelination,^23^ the average CVS+ rate assessed in this population varies significantly, ranging from 12% to 78%.^20,21^ This variability could be related, at least partially, to technical differences in CVS assessments, but it is also possible that some of these differences may be related to underlying unknown pathologic factors. For instance, if some of the MRI changes detectable in MOGAD acutely are related to transitory phenomena rather than perivenular demyelination, the assessment of CVS in this population can be significantly affected by the time at which imaging is acquired in relation to the acute clinical presentation.

Our study did not identify any PRL in the MOGAD cohort. PRLs have been described as biomarkers of chronic active lesions in MS, characterized by a paramagnetic rim surrounding nonenhancing chronic lesions, indicative of iron-laden microglia at the lesion edge.^16^ These lesions have been associated with worse clinical outcomes and seem to be relatively specific to MS, having been reported in around 7% of other diseases in CNS.^12^ Although our sample size is limited, these findings may prompt further investigation into the role of activated microglia in MS pathology compared with MOGAD, which seems to be more distinct from the CVS.

All this considered, this study highlighted some specific features of CVS and PRLs in MOGAD imaging. CVS is a useful imaging sign for differentiating MS from other CNS inflammatory diseases.^25,34^ However, several factors must be considered in MOGAD. First, owing to the high rate of tumefactive lesions^35^ and the common involvement of infratentorial structures,^17,36,37^ a nonnegligible proportion of lesions is not suitable for CVS analysis. Second, the rate of CVS+ lesions in MOGAD is influenced by the timing of MRI acquisition, with twice as high values in remission compared with acute MRIs. This has implications for the identification of an optimal cutoff for MS diagnosis but also for the interpretation of the high variability of findings in MOGAD literature. PRLs have not been detected in our pediatric MOGAD cohort, while they have been described in pediatric MS, thus suggesting they can be a useful radiologic marker in the differential diagnosis between these 2 diseases.

## Glossary

CVS: central vein sign
EDSS: expanded disability status scale
FLAIR: fluid-attenuated inversion recovery
IQR: interquartile range
IRB: institutional review board
MOGAD: myelin oligodendrocyte glycoprotein antibody-associated disease
MS: multiple sclerosis
POMS: pediatric-onset MS
PRL: paramagnetic rim lesion
SBI: susceptibility-based imaging
WM: white matter
